# Microbeam X-Ray Investigation of the Structural Transition from Circularly Banded to Ringless Dendritic Assemblies in Poly(Butylene Adipate) Through Dilution with Poly(Ethylene Oxide)

**DOI:** 10.3390/polym17152040

**Published:** 2025-07-26

**Authors:** Selvaraj Nagarajan, Chia-I Chang, I-Chuan Lin, Yu-Syuan Chen, Chean-Cheng Su, Li-Ting Lee, Eamor M. Woo

**Affiliations:** 1 Department of Chemical Engineering, National Cheng Kung University, Tainan 701-01, Taiwan; nagarajan.tech@gmail.com (S.N.); mike19961103@yahoo.com.tw (C.-I.C.); jane880311@gmail.com (I.-C.L.); ju02091@gmail.com (Y.-S.C.); 2 Department of Chemical and Materials Engineering, National University of Kaohsiung, Kaohsiung 811726, Taiwan; ccsu@nuk.edu.tw; 3 Department of Materials Science and Engineering, Feng Chia University, Taichung 407-24, Taiwan

**Keywords:** poly(butylene adipate), lamellar self-assembly, synchrotron radiation, microbeam X-ray analysis, scanning electron microscopy, structural coloration

## Abstract

In this study, growth mechanisms are proposed to understand how banded dendritic crystal aggregates in poly(1,4-butylene adipate) (PBA) transform into straight dendrites upon dilution with a large quantity of poly(ethylene oxide) (PEO) (25–90 wt.%). In growth packing, crystal plates are deformed in numerous ways, such as bending, scrolling, and twisting in self-assembly, into final aggregated morphologies of periodic bands or straight dendrites. Diluting PBA with a significant amount of PEO uncovers intricate periodic banded assemblies, facilitating better structural analysis. Both circularly banded and straight dendritic PBA aggregates have similar basic lamellar patterns. In straight dendritic PBA spherulites, crystal plates can twist from edge-on to flat-on, similar to those in ring-banded spherulites. Therefore, twists—whether continuous or discontinuous—are not limited to the conventional models proposed for classical periodic-banded spherulites. Thus, it would not be universally accurate to claim that the periodic circular bands observed in polymers or small-molecule compounds are caused by continuous lamellar helix twists. Straight dendrites, which do not exhibit optical bands, may also involve alternate crystal twists or scrolls during growth. Iridescence tests are used to compare the differences in crystal assemblies of straight dendrites vs. circularly banded PBA crystals.

## 1. Introduction

Spherulites have been known to be packed via crystal-by-crystal aggregation into a final entity that may assume many different morphological forms, which is somewhat similar to how nature’s hexagonal ice crystals self-assemble into snowflakes that can take endless patterns [1,2,3,4,5]. However, debates persisted for a long time for polymer spherulites [6,7,8,9,10,11,12,13,14,15,16,17,18,19], such as poly(ethylene adipate) (PEA) [8,20,21,22,23,24] or polyethylene (PE) spherulites, etc. [25,26,27,28,29,30,31,32,33,34,35,36]. Helical structures present in nature, such as the double-helix conformation of DNA, were first characterized in detail in the early 1950s [37,38,39]. Building upon this model, numerous researchers have proposed mechanisms such as screw dislocations [40,41] or twists induced by thermal or mechanical fields [34,42]. Subsequently, some speculated that helix twisting in polymer systems could result from unbalanced surface stresses associated with chain folding in lamellae [43,44,45,46]. The speculation emerged from the notion that polymer lamellae might adopt conformations resembling continuous helices or helicoidal structures, akin to the well-established, long-range double-helix conformation of DNA. Yet, by using a variety of polymeric assemblies, newer and more evidence-justified mechanisms were proposed to exemplify that periodic bands are not necessarily constructed by continuous helix-twist lamellae; instead, periodic-interfaced lamellae with discontinuity are feasible for the noted optical banding patterns [47,48,49,50,51,52,53,54]. Such views are universal, covering a wide range of different polymers. As an example, Tu and Woo et al. [49], by using a model of poly(nonamethylene terephthalate) (PNT), have exemplified that the interior fan-like lamellae are not continuous but grow discontinuously in a fractal pattern to evolve branches expanding outward and upward, finally taking a fan-like pattern, with discontinuous interfaces in each of the growth cycles. A PLLA blend system was prepared [50], from which ring-banded PLLA was crystallized at T_c_ = 110 °C; the authors thus proved that the interior PLLA lamellae are arranged in a discontinuous pattern. In the 3D bulk, edge-on and flat-on lamellae are self-assembled mutually to form a grating corrugate-board architecture that is responsible for the periodic optical rings, as viewed in POM images. This contrasts with the conventional model, in which the periodic optical birefringence bands in polymer crystals are thought to result from a single crystalline plate (similar to a DNA molecule) undergoing continuous helicoidal twisting. In crystal aggregation during growth, lamellae do occasionally twist or scroll to fit into a jammed space; however, it is thermodynamically non-feasible that they maintain the continuity and pitches of twist for an uninterrupted length (~500 μm in radius) till the periphery of spherulites. A lamella is a single crystal; by nature, it takes a micrometer dimension of ca. 3 μm in width and thickness ~10–20 nm in thickness. It is not kinetically possible that a single crystal (lamella) can grow uninterruptedly to ~500 μm in length to undergo “continuous helix twist” inside a polymer spherulite. Later on, numerous studies [55,56,57] using synchrotron-radiation microbeam X-ray diffraction investigations further proved that such discontinuous assemblies are valid. That is, microscopy and polycrystalline lamellae with a cross-hatching growth are responsible for the periodic bands observed in POM characterization.

Poly(1,4-butylene adipate) (PBA) is a biodegradable aliphatic polyester; however, its molecular chains do not possess H-bonding or tough chains, such as benzene rings, in its chemical structure, leading to rather weak mechanical strength and a low melting point of PBA. In application, because of the low strength of PBA, it is rarely used by itself; it is usually incorporated into commercial plastics or copolymerized with other monomers to form commercial copolymers. A block copolymer based on PBA and poly(1,4-butylene succinate) (PBSu), commercially produced by Showa-Denko Corporation, is widely used in the applications of biodegradable packaging films or plastic bags [52]. PBA has been widely studied regarding its crystalline polymorphic phases with α-form and β-form lattices [37,38,39] when crystallized at various temperatures. Other abundant related studies dealt with the higher hierarchical levels of morphology of the PBA spherulites [25,58,59,60,61,62,63].

PEO has been found to be an effective diluent and, therefore, does not influence the final crystal patterns of PBA [63]. In line with the previous work, this investigation further expanded the interfaces between the periodic assembly by highly diluting PBA during crystallization in the presence of PEO. Powerful synchrotron-radiation wide-angle and small-angle X-ray diffraction (WAXD and SAXS) techniques were used for discerning the crystals’ assembly in micro-domains. Standard microfocal techniques were able to produce a microbeam of 6 μm in size. Scanning electron microscopy (SEM) characterization and polarized-light optical microscopy (POM) were used to investigate the morphology. By manipulating the dilution of PBA with PEO across a wide range of concentrations (from 25 to 90 wt.%) in PBA/PEO blends, the objective of the study was to control the PBA’s crystalline patterns, which, at the same T_c_, could be modulated from circular-smooth ring bands to straight dendrites with completely different lamellar geometry. This technique of diluent modulation made it possible to assess the effects of lamellar twist on periodic bands vs. straight dendrites in spherulitic aggregates.

## 2. Experimental

### 2.1. Materials and Preparation

**Scheme 1:**
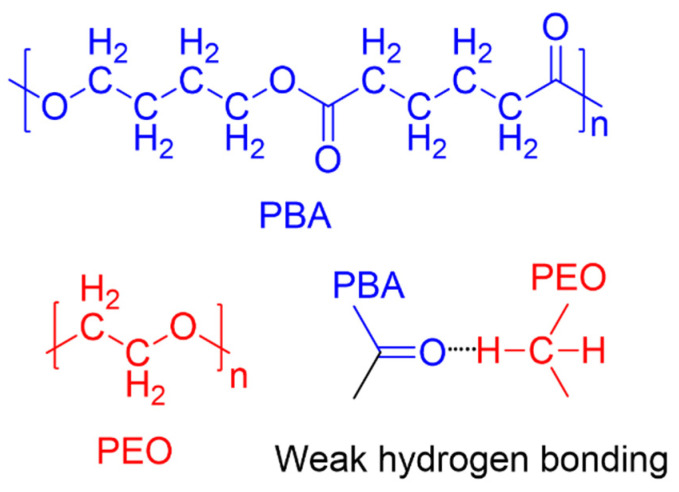
Chemical structures of PBA and PEO and possible weak hydrogen bond interaction between electron-rich carbonyl oxygen (C=O) from PBA and electron-poor C–H groups from CH_2_ in PEO.

PBA was supplied by Aldrich Chemicals Company, Inc. (St. Louis, MO, USA), with M_w_ = 12,000 g/mol, T_g_ = −66.2 °C, and T_m_ = 56~60 °C. PEO, also supplied by Aldrich Chemical Company, Inc. (USA), has M_w_ = 200,000 g/mol, T_g_ = −60 °C, and T_m_ = 64 °C. Polymers were dissolved in chloroform and etched with methanol solvents purchased from Aldrich Chemicals Company, Inc. (USA). Four mixture compositions were prepared: PBA/PEO (wt./wt.) = 75/25, 50/50, 25/75, and 10/90, respectively, where PBA was highly diluted by the miscible PEO constituent (25–90 wt.%). Well-stirred polymer solutions containing 4 wt% of PBA and PEO (in chloroform) were cast onto glass slides at 35 °C to form thin films. The solvent was allowed to evaporate under ambient conditions, followed by drying in a vacuum oven. The dried samples were then melted at 150 °C, held for 2 min, and subsequently crystallized at specific crystallization temperatures (T_c_). The chemical structures of PBA and PEO, along with their potential hydrogen bonding interactions, are illustrated in Scheme 1. To enhance the visual contrast of the spherulitic morphology, the crystallized samples were etched with methanol to remove the PEO component from the spherulites selectively.

### 2.2. Apparatus

*Polarized-light optical microscopy (POM):* This was performed using Nikon Optiphot-2, Tokyo, Japan. POM, equipped with a Nikon Digital Sight (DS-U1) for in situ recording along with a CCD digital camera (NFX-35). A microscopic heating stage (Linkam THMS-600 with a T95 temperature programmer, Surrey, UK) was used for temperature control. For birefringence coloration, a λ-tint plate (530 nm) was used in POM.

*High-resolution field-emission scanning electron microscopy (HR-FESEM):* Hitachi SU8010 (HR-FESEM) Tokyo, Japan was used for examining the detailed top surface and interior morphology. The specimens were sputter-coating using platinum.

*Iridescence Observation:* The crystallized sample (~1.8 cm × 1.8 cm) was placed on a glass slide and positioned directly below a white flashlight on a black background as the light source. Photographs were taken from the top view in a dark room, with the light shining perpendicular to the sample surface. This setup allowed clear observation of iridescence arising from internal grating structures within the spherulites.

*Synchrotron-radiation wide-angle X-ray diffraction (WAXD) and small-angle X-ray scattering (SAXS):* Powerful X-ray radiation was provided by National Synchrotron Radiation Research Center (NSRRC, located in Hsinchu, Taiwan). Parameters of the radiation source and facilities were as follows: photon energy: 15 keV, wavelength: 0.8263 Å, and q-range: 0.1~3 Å^−1^. IU22 detector: Dectris Eiger 1M, Baden-Dättwil, Switzerland with a detector distance of 0.082 m. Microbeam size was ca. 6 μm. For microbeam X-ray diffraction in synchrotron-radiation facility, thin-film specimens were prepared via solution-casting onto a substrate of polyimide films (PI or Kapton for transparency to X-rays). Procedures were as follows: A droplet of the solution (4 wt.%) was cast on the PI film, spread uniformly, and the solvent was left to evaporate in air, followed by placing it in a vacuum oven for one day. The dried specimens were then subjected to predetermined heat treatments to fully crystallize at specific T_c_. Methanol was used as an etching agent to remove the PEO constituent from the crystallized (PBA/PEO) films in order to avoid the crystalline peaks from PEO. The crystallized and etched specimens were set up in holder of synchrotron X-ray microbeam. The beam stopper was carefully positioned so that both WAXD and SAXS signals could be simultaneously recorded in one scan; subsequently, both sets of data were later analyzed using on-site software. Both 1D and 2D X-ray diffraction data were gathered by step-moving the microbeam stepwise at specific microdomain spots of the periodically banded spherulites in the specimens.

## 3. Results and Discussion

### 3.1. Assembly Patterns in Highly Diluted PBA/PEO Blends

**Figure 1:**
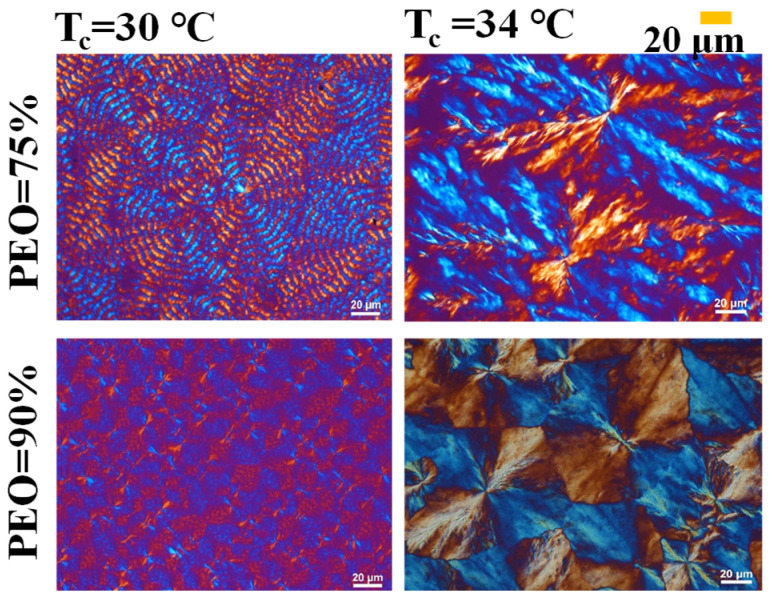
A matrix of composition and temperature showing POM micrographs for highly diluted PBA/PEO (PEO = 75 wt.%) and (PEO = 90 wt.%), crystallized at T_c_ = 30 or 34 °C, respectively, by quenching from same T_max_ = 180 °C.

Methanol or water could be used as a differential solvent for selectively etching out the PEO constituent, leaving the main PBA crystal assembly intact. Effects of various T_max_ on the PBA morphology crystallized at the same T_c_ = 30 °C were investigated and reported in our earlier work [63]. From these results, T_max_ = 180 °C for melting PBA specimens prior to quenching for isothermal crystallization was found to be the most appropriate. Figure 1 shows the PBA morphologies in the PBA/PEO blend of two compositions (75/25 and 10/90) crystallized at two different T_c_’s (after being melted at T_max_ = 180 °C). The blue slanted line marks the border between regular vs. ringless patterns. At the same T_c_ = 30 °C, with the PEO content increasing from 75 to 90 wt.%, the birefringence patterns change from regularly circular bands to completely irregular patterns, suggesting that the lamellae in the PBA aggregates self-assemble from regular to irregular periodicity with T_c_ varying from 30 to 34 °C. At the higher T_c_ = 34 °C, both compositions display straight dendritic patterns, and no circular rings are seen at all. Obviously, only two opposite morphology patterns are present, i.e., circularly banded rings or straight dendrites. By adjusting either the composition or the crystallization temperature, the birefringence patterns of the PBA/PEO blend can be modulated from regular banded to straight dendritic morphology.

**Figure 2:**
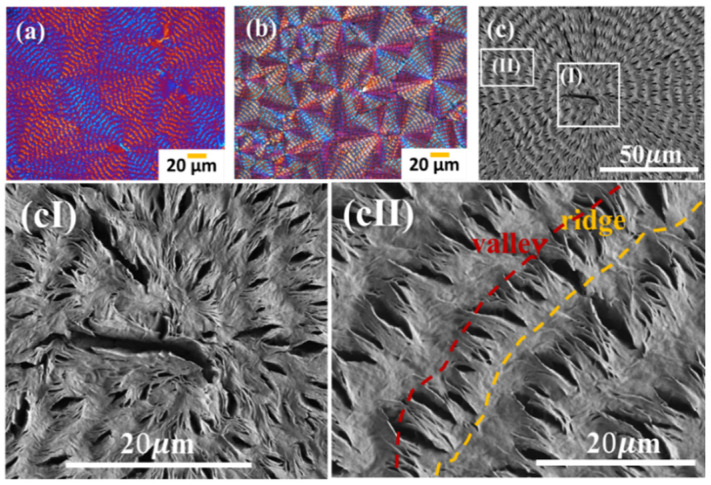
(**a**,**b**) POM (pristine vs. etched); (**c**) SEM image of entire top surface of spherulites in PBA/PEO (75/25) crystallized at T_c_ = 30 °C; (**cI**) zoom-in of banded region and (**cII**) nucleus regions.

Figure 2a,b show POM micrographs, with Figure 2a representing the pristine specimen and Figure 2b showing the solvent-etched specimen for comparison. Figure 2c shows the SEM micrographs of the top surface of the nucleus region and the banded region far from the nucleus, respectively, for PBA/PEO (75/25) at T_c_ = 30 °C. Figure 2(cI) is a magnified image of the nucleus shown in Figure 2c, and Figure 2(cII) shows the banded region far from the nucleus. From the low-magnification SEM graph of Figure 2c, the lamellae seem to twist “continuously” from one cycle to the next cycle of growth by radiating outward from the nucleus. However, the top surface may be misleading to such a superficial concept. The zoomed-in SEM graph in Figure 2(cII) shows the end of a growth cycle in greater detail, marked with a yellow-colored circumferential dashed line. The original radially oriented edge-on lamellae (on the ridge) twist and bend at a 90° sharp angle to merge into the tangential direction to form a valley. In other words, these radially oriented edge-on lamellae, though twisted, do not continuously extend into the next growth cycle. Perhaps, one may argue that the top-surface morphology might not be sufficient to come to such a conclusion. Yet, in the following section, using an interior 3D dissection technique of SEM microscopy, it can be demonstrated conclusively that the lamellae in repetitive cycles are actually non-continuous, i.e., there are interfaces due to periodically mutual perpendicular intersections between the successive cycles of growth.

**Figure 3:**
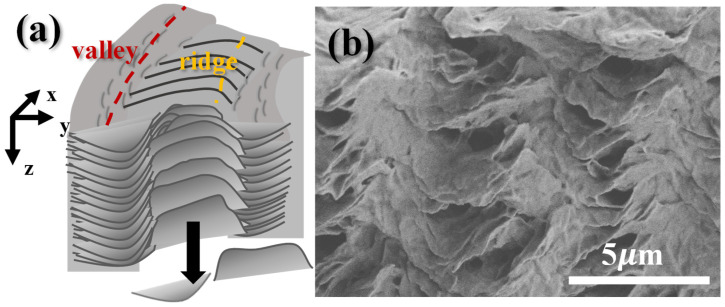
(**a**) Scheme for a single pitch of periodic assembly covering valley–ridge and (**b**) SEM of fractured interiors of a regularly banded region of PBA/PEO (75/25) at T_c_ = 30 °C.

Figure 3 shows a scheme (Figure 3a) for the magnified details of the interior assembly and an SEM graph for the fractured interior of the banded region of PBA/PEO (75/25) crystallized at T_c_ = 30 °C, which possesses a circularly ring-banded pattern, just like that seen in neat PBA (with no PEO) [63]. Figure 3b shows that the interior lamellae underneath the valley zone are all flat-on oriented (circumferentially oriented) with respect to the graph paper; by contrast, the interior lamellae underneath the ridge zone are all of “edge-on” orientation (i.e., normally oriented) with respect to the plane. The ridge’s edge-on lamellae collectively twist and bend at a 90° angle to collapse and mutually stack in the circumferential direction, where they all become flat-on oriented. Nevertheless, these lamellae are not continuous from nucleus to periphery, but there are distinct periodic interfaces between the radially oriented lamellae and the tangentially oriented ones, clearly testifying to discontinuity.

### 3.2. Assembly of the Interior vs. Top Surface of Circularly-Banded PBA Crystal Aggregates

**Figure 4:**
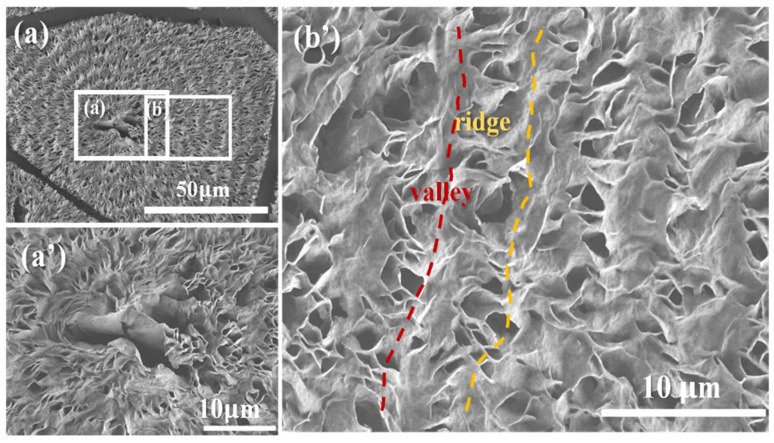
(**a**) Top-surface SEM micrograph; (**a’**) zoom-in of the nucleus region; (**b’**) zoom-in of the periodic bands away from the nucleus for top surface of periodic-banded PBA spherulites from highly PEO-diluted (PBA/PEO = 50/50) blends crystallized at T_c_ = 30 °C.

As the PEO content is further increased to 50% (PBA/PEO = 50/50 wt./wt.), the ring bands start to be corrupted. Assembly analysis is then focused on a ring-banded PBA spherulite crystallized from another highly PEO-diluted (PBA/PEO = 50/50) blend at the same T_c_ = 30 °C. Figure 4a shows SEM micrograph at low magnification for viewing an entire spherulite, Figure 4a’ a zoom-in SEM micrograph for the nucleus center of the water-etched specimen, and Figure 4b’ periodic bands away from the nucleus region for the top surface of periodic-banded PBA spherulites crystallized from a highly PEO-diluted (PBA/PEO = 50/50) blend at T_c_ = 30 °C. The nucleus center displays typical tri-branch cracks, and circular rings start immediately outside the nucleus region (Figure 4a’). The ridge and valley zones on the top surface show alternate edge-on plates (ridge) and flat-on plates (valley) in cycles at a fixed pitch, where crevices are seen only on the ridge zone and never on the valley zone. It is easy to understand that the lamellae on the ridge are edge-on oriented (crystal plates being oriented vertically to the substrate plane) and the crevices are the inter-lamellae interfaces that are expounded upon by water-etching on the specimens. In Figure 4b’ showing the zoomed-in SEM graph, the yellow lines on the top surface label the marks where the frontiers (i.e., tips of ridge zones) of ridge zones are located. Underneath these yellow dashed lines on the top surface, the lamellae in the interiors are separated by parallel crevices.

**Figure 5:**
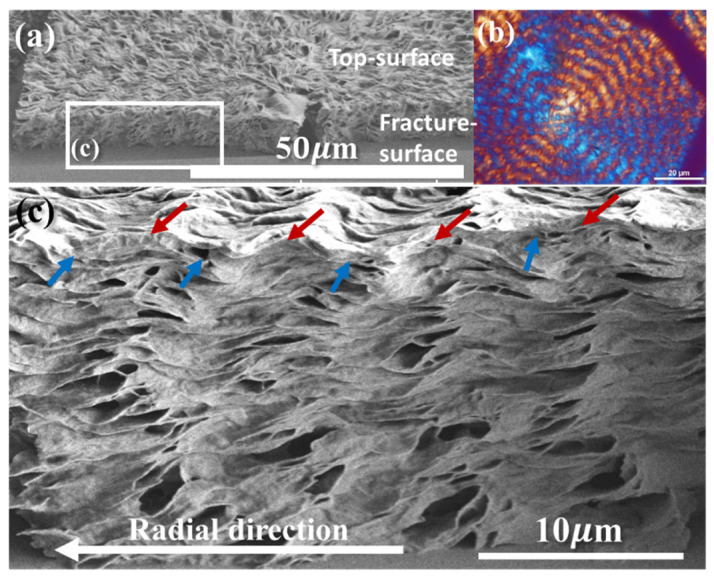
(**a**) Full-view SEM images of top surface vs. dissected interior bands revealing interior alternate lamellae as ridge and valley, with (**b**) POM graph with scale bar 20 µm and (**c**) SEM image showing dissected interior bands for ring-banded PBA spherulites from highly PEO-diluted (PBA/PEO = 50/50) blend crystallized at T_c_ = 30 °C. [red arrows: ridge zone; blue arrows: valley zone].

Figure 5a shows a full view of the interior lamellar assembly, which is almost identical to that of the top surface, except that there exists an offset between the interior and top-surface assembly. Figure 5b is the corresponding POM for the periodic bands, and Figure 5c is a zoomed-in SEM image showing the details of assembly. There exists an offset between the topology and interior cross-section morphology, and the crevices on the topology are offset by a displacement from those in the interior cross-section. Thus, the top-surface crevices display a fixed offset from those in the cross-section, and the offset distance (ca. ~3 μm) is exactly equal to a half-pitch of the PBA inter-band spacing (6 μm). This is to say that the assembly pattern on topology is opposite to those in the interior assembly, offset by a fixed distance due to an interior-to-top transition. The experimental fact also suggests that analyses of periodic bands should not be confined or based only on the topology of thin films, which may mislead the interpretation due to the offset non-matching between the superficial topology and interior cross-section. Three-dimensional views, by dissecting into inner crystal architectures, are essential for obtaining complete pictures for correlating the outer and inner assemblies.

### 3.3. General Features of Periodically Circular Assembly as Viewed from Interiors

**Figure 6:**
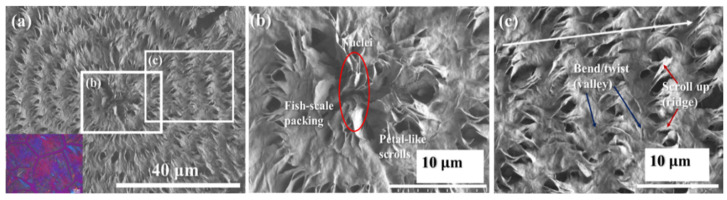
(**a**) Top-surface SEM graphs of PBA/PEA inset with POM, (**b**) Zone I nuclei region with marked red circle, and (**c**) Zone II periodic-banded zone away from nucleus of ring-banded PBA/PEO (wt./wt. = 25/75) crystallized at 29 °C. Long white arrow indicates the radial direction.

As the PEO content is further increased to an even higher content of 75 wt.% (i.e., PBA/PEO = 25/75), the PBA component (accounting only for a quarter fraction of the mixture) now becomes highly diluted, and the PBA morphology is dramatically different. Circularly banded patterns could no longer be maintained, especially at high T_c_ > 30 °C. Figure 6a shows the SEM graphs for an entire periodic-banded PBA/PEO (25/75) spherulite crystallized at 29 °C, where two zones have been zoomed in for analyses. The lamellae in PBA spherulites crystallized at T_c_ = 29 °C from a highly diluted PBA/PEO blend (75 wt.% PEO in the PBA/PEO mixture) are no longer circular bands but become straight dendritic, as shown in the zoomed-in images in Figure 6b,c. Figure 6b illustrates the region near the nucleus, where the crystal plates are sheaf-like and mostly edge-on oriented, pointing in two opposite directions. Growing normally to the sheaf-crystal plates are the eye-like zones, where the crystal plates are flat-on and self-assemble as “fish scales” stagger-stacks. These flat-on “fish-scale” stacked crystals do not remain like this for a long period before they collectively scroll into edge-on orientation (marked with flower-petal-like scrolls in Figure 6a). Figure 6c illustrates the regions of periodic bands farther away from the nucleus center. Clearly and alternately, the scroll-up petal-like zone of the edge-on crystal plates is followed by flat-on fish-scale stack crystal plates. The main feature is the periodic tail-bending/twisting of the lamellae into valleys, where they pack into fish-scale-like, flat-on stacks in the interiors; meanwhile, the lamellae scroll up to form the ridge band (vertically oriented in the interiors).

**Figure 7:**
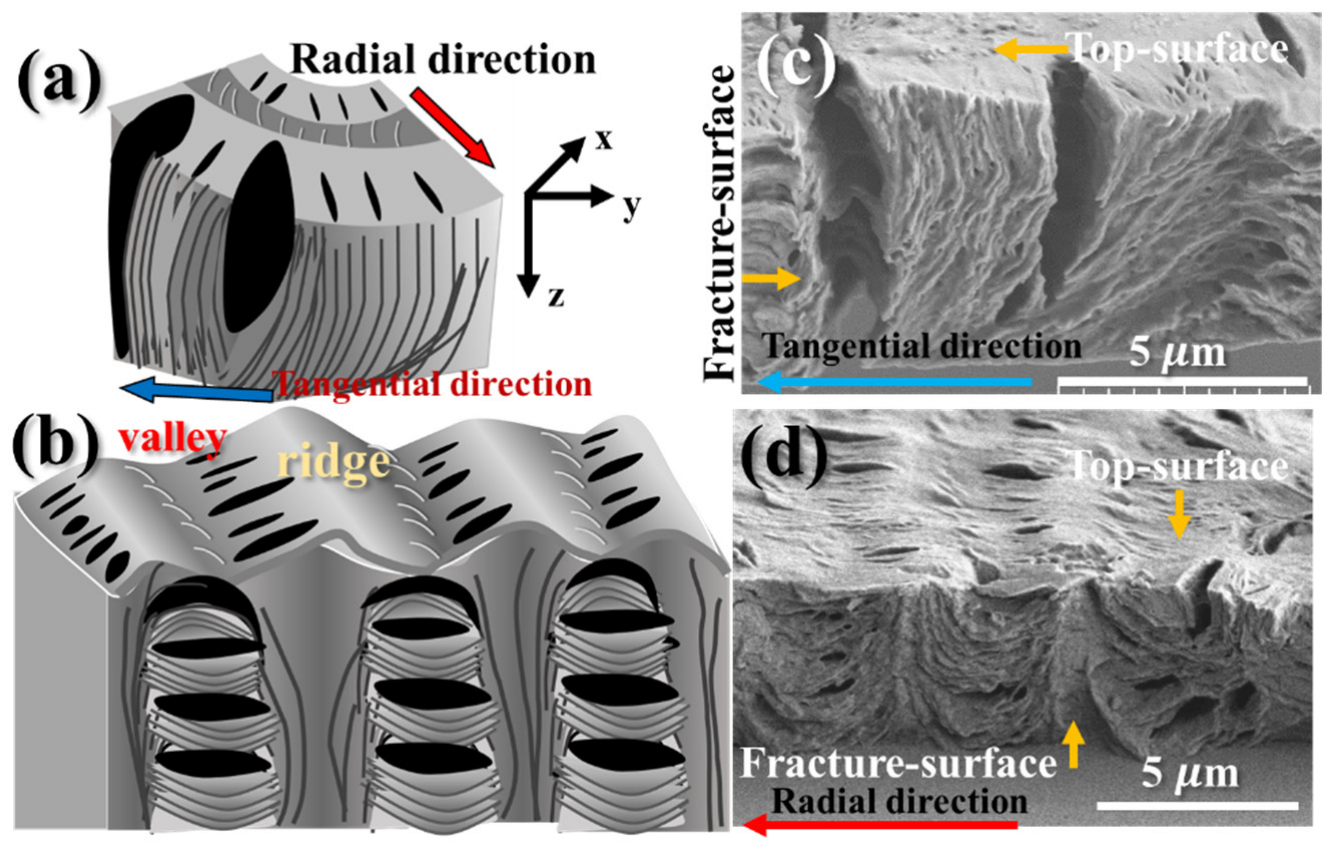
General assembly features of periodic circular-banded PBA: (**a**) scheme for circumferential dissection, (**b**) scheme for radial dissection, (**c**) SEM graph for interior normal-oriented lamellae underneath a ridge zone, and (**d**) SEM graph for interior alternate horizontal vs. normal-oriented lamellae underneath ridge-valley along a radial dissection line.

Figure 7a,b show the schemes for fractured interiors along the circumferential direction (Figure 7a) and the radial direction (Figure 7b), respectively. Figure 7c,d show the experimental SEM evidence for the interiors cut along these two directions, respectively. The schemes are an exemplification of the key features of the interior grating assembly, but they are fully supported by the experimental data.

### 3.4. Micro- to Nano-Views on Assembly in Straight-Dendrite PBA Aggregates

**Figure 8:**
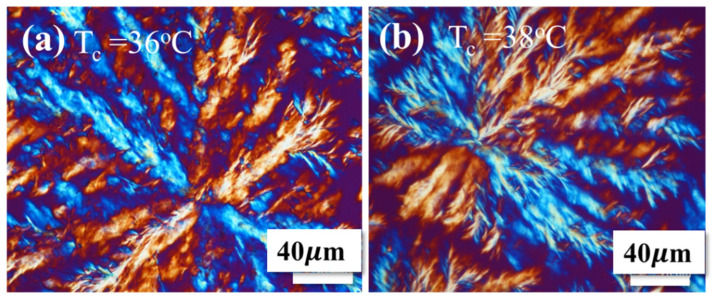
POM graphs of straight dendritic PBA spherulite from PBA/PEO (25/75) blend crystallized at high T_c_: (**a**) 36 °C and (**b**) 38 °C, where the PBA spherulites are all straight dendritic and non-circular-banded.

As PBA is highly diluted to crystallize from PBA/PEO = 25/75 at higher T_c_’s (>30 °C), circular patterns are no longer maintained; instead, crystal self-assembly always leads to straight dendrites. Figure 8 shows the POM graphs for dendritic PBA spherulites crystallized from a highly diluted PBA/PEO (25/75) blend at two high T_c_’s = (Figure 8a) 36 °C and (Figure 8b) 38 °C, respectively. No periodically circular bands are seen at all; instead, all these ringless PBA spherulites are composed of straight dendritic (with no circular rings) lamellae, displaying a straight dendritic pattern, without any circular bands. Crystallization at these high temperatures of 36 and 38 °C led to variation in the coarseness of the lamellae that are packed in the straight dendritic aggregates. These POM images yield only general views of crystal assembly; more detailed SEM results and analyses are to be discussed in following sections.

**Figure 9:**
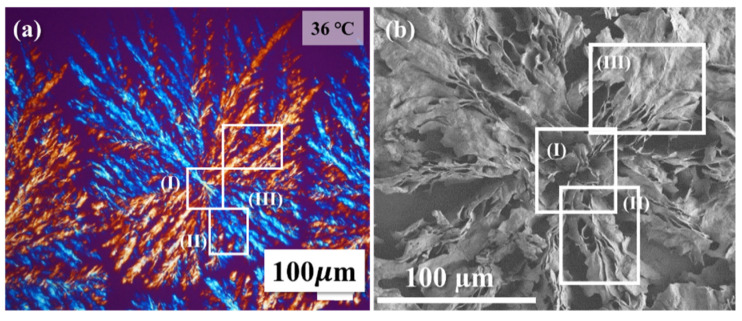
(**a**) POM and (**b**) corresponding SEM graph for methanol-etched top surface of PBA/PEO (25/75) crystallized at T_c_ = 36 °C, with highly straight dendritic patterns.

The non-banded and highly straight dendritic PBA spherulites are further analyzed for the details of assembly. Figure 9a,b show the POM and SEM graphs, respectively, for the methanol-etched top surface of PBA/PEO (25/75) crystallized at 36 °C. Note that at T_c_ = 36 °C, the bands are mostly corrupted, and the optical patterns reveal highly dendritic lamellae. Three zones were zoomed in for analyses: Zone (I), nuclei; Zone (II), main stalks of dendrites; and Zone (III), tail-ends of lamellae, where the initial edge-on lamellae rapidly collapse into flat-on orientation. Figure 9b shows an SEM image of the entire dendritic PBA spherulite at lower magnification to cover several different zones of lamellar assembly, where three micro-domains (Zone I, II, and III) have bene zoomed in for focal analysis. The corresponding zones are marked as I, II, and III, respectively, on the POM micrograph for identifying the respective birefringence patterns (Figure 9a).

**Figure 10:**
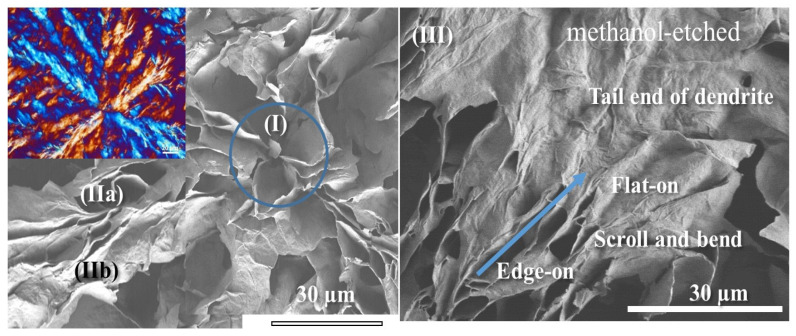
Zoom-in stacked SEM micrographs showing Zone I—nucleus on top surface; Zone IIa—edge-on lamellae; Zone IIb—flat-on lamellae of main dendrite-stalks on top surface; and Zone III—twisting/scrolling of lamellar ends transitioning from edge-on to flat-on orientations as they grow away from nucleus in PBA/PEO (25/75) crystallized at 36 °C. [Inset to the upper-left corner: POM micrograph for the dendritic patterns].

The straight dendritic PBA spherulites are unique. Upon closer examination of the assembly of PBA/PEO (25/75) crystallized at T_c_ = 36 °C, the straight dendrites are composed of several main stalks packed with loosely leaf-like branches, as shown in Figure 10. Three distinct zones (I, IIa, and IIb, respectively) of interest are zoomed in for in-depth analyses. Zone I shows that the nuclei-sheaf crystals are mostly edge-on (with the crystal plates orienting normal to the substrate), as they are tiny and crowded at the nucleus center. Such a compact packing mode allows them to be efficiently assembled in a limited space. Zone IIa shows that most of the lamellae are edge-on oriented, although some of them still twist randomly to become locally flat-on (Zone IIb). From the SEM graphs of Figure 10, it is easy to discern that although the length and width dimensions of these crystal plates are ca. 5–10 μm, the thickness of these scrolled-up crystal plates is sub-micro at tens or hundreds of micrometers. The dissected interior morphology clearly indicates that the lamellae (single crystal plates) are not all straightly “flat-on” but scroll, bend, and twist irregularly or incidentally. Without analyses via interior 3D dissection techniques, the top surfaces of either straightly dendritic or circularly banded crystals would not provide sufficient and critical evidence to elucidate the assembly in the entire bulk.

The zoom-in of Zone III, showing the tail ends (Zone III) of the straight dendritic lamellae, is further exemplified clearly for interior assembly. The originally edge-on lamellae in the radial growth collapse into broad-leaf-like platelets that obviously are flat-on oriented. Note that the PBA/PEO (25/75) spherulite at high T_c_ = 36 °C is definitely non-banded but dendritic. This morphological evidence clearly indicates that lamellae may twist not just in banded spherulites but also in straight dendritic ones. Obviously, near the nuclei, the lamellae are much more jammed and crowded, and they naturally pack in edge-on orientations during the initial stage. Subsequently, as the lamellae fan outward, farther from the nuclei, the space expands significantly, and thermodynamic factors drive them to become broad-leaf-like flat-on plates in order to branch out and to occupy the expanding space. Obviously, the crystal-plate twists from edge-on to flat-on could take place in the straight dendritic spherulites (along the blue-arrow direction), suggesting that the lamellar twists are not limited to the conventional periodic-banded ones. Thus, it would not be universally applicable to claim that the periodic circular bands found in polymers or small-molecule compounds are caused by continuous lamellar helix-twists, as, obviously, straight dendrites without showing any optical bands may also involve such alternate crystal twists or scrolls in growth. In these straight dendrites, occasional scrolls and bending are also common, as marked on the bottom-right corner of the SEM micrographs.

**Figure 11:**
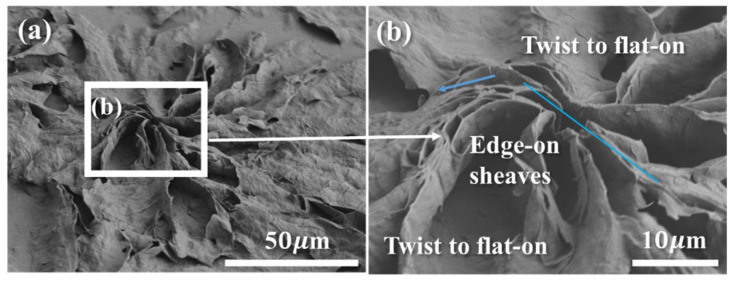
SEM micrographs for (**a**) nucleus, showing edge-on straight crystal sheaves, and (**b**) a magnified view of squared-b region showing crystal plates’ gradual twist to flat-on (parallel to substrate) on top surface in PBA crystallized from PBA/PEO (25/75) at T_c_ = 36 °C.

The crystal-plate twist from edge-on to flat-on orientations is not confined to the zone of straight dendrites’ tails; such twists may also take place while the lamellae start to grow away immediately from the nucleus center. Again, for the straight dendritic lamellae in PBA (PBA/PEO = 25/75) crystallized at T_c_ = 36 °C, Figure 11 shows the SEM micrographs of (Figure 11a) the nucleus region, where edge-on straight crystal sheaves can be observed, and (Figure 11b) a magnified view of the squared-b block, showing the crystal plates gradually twisting from an edge-on to a flat-on orientation (parallel to substrate) on the top surface.

### 3.5. Synchrotron Microbeam X-Ray Analysis on Straight Dendritic vs. Circular-Banded Zones

**Figure 12:**
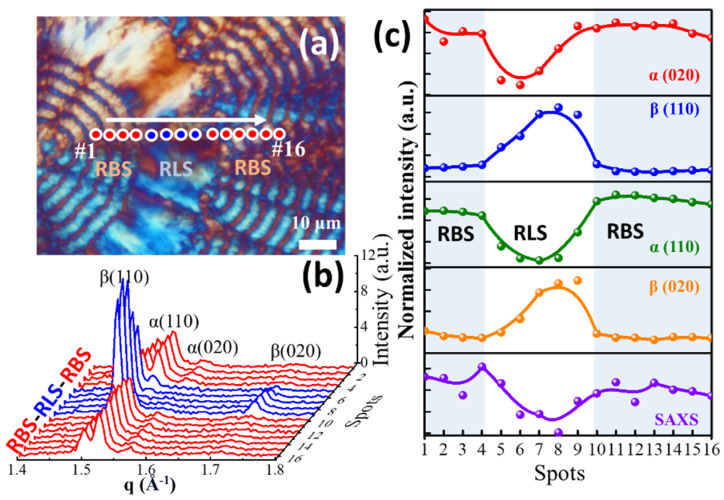
(**a**) Optical micrograph for PBA crystallized alternatively with successive zones of ring-banded spherulite (RBS) vs. ringless spherulite (RLS), (**b**) microbeam X-ray WAXD 1D profiles, and (**c**) microbeam X-ray WAXD on 16 spots across alternate RBS and RLS zones. Spots #1–4: ring-banded; Spots #5–9: ringless; Spots #10–16: ring-banded (#1–16: one complete growth cycle).

To further support the morphology analyses of SEM results, orientations of the lamellae in circular banded vs. ringless dendrites of PBA spherulites were characterized using synchrotron microbeam X-ray diffraction. An analysis of polymorphism and crystal lattice structure reveals their influence on the morphological transition from RBS to RLS zones in PBA-RBS, as demonstrated by POM morphology and X-ray diffraction analyses. Figure 12a is a micrograph of PBA crystallized alternatively to display successive ring-banded and ringless zones, which were intended for an X-ray microbeam to focus on the alternate zones for comparison. In the radial direction of a PBA spherulite, 16 spots of microbeam focuses were marked from #1 to #16 to traverse stepwise (once every 1 μm) from the ring-banded zone (RBS) to the ringless one (RLS, with straight dendrites). Within a temperature window, the typical crystallization of PBA expectedly results in the formation of a stable α-crystal structure, which is distinguished by its monoclinic unit cell parameters: a = 0.67 nm, b = 0.80 nm, c = 1.42 nm, and β = 45.5°. This α-crystal structure is associated with the P21/n space group, indicative of its precise symmetry and molecular organization. By contrast, the β-form crystal may also co-exist with the α-form, and the β-form crystal presents an orthorhombic unit cell structure, characterized by dimensions of a = 0.505 nm, b = 0.736 nm, and c = 1.44 ± 0.05 nm, featuring a distinct planar zigzag conformation [64,65]. Despite the predominant formation of the α-crystal structure under typical conditions, variations in the high-temperature crystallization environment may induce the emergence of β-form crystals. The variation of crystallization temperatures from 32 °C to 40 °C and back to 32 °C notably impacts the spherulitic morphology, transitioning between RBS and RLS structures. Additionally, precise microbeam targeting enables a more accurate determination of polymorphism at specific morphological sites. Figure 12b shows PBA crystallized at 32 °C; the morphology of PBA-RBS prominently features the α-crystal structure, characterized by distinct peaks corresponding to (110) and (020). Conversely, at T_c_ = 40 °C, the RLS structures predominantly comprise β-form crystals, evidenced by prominent signals of (110) and (020). Upon reaching 32 °C again, the RLS structures transition back to the α-form, aligning with the crystalline arrangement observed in the RBS morphology. Figure 12c compares the WAXD signal intensities for the crystal unit-cell orientation changes and SAXS signals for lamellae orientation transitions (as shown in Figure S1). These analyses confirm the transition of lamellar alignment in alternating zones. Figure S2 provides quantitative insight into the long-period parameters of PBA-RBS. The long period (L_o_) was determined to be 10.8 nm, with a crystal thickness (L_c_) of 4.6 nm and an amorphous thickness (L_a_) of 6.2 nm. The overall crystallinity was measured as 42.5%. By such comparison of both WAXD and SAXS data from simultaneous microbeam characterization, a more comprehensive insight can be gleaned. The discernible presence of SAXS signals in the RBS and their conspicuous absence in the RLS region may be ascribed to the distribution of flat-on lamellae that are prevalent across the RLS region. This intriguing behavior highlights the dynamic interplay between temperature and polymorphic transitions. Consequently, the valleys of the RBS and RLS regions are likely dominated by β-forms, while the ridge regions are enriched with α-forms, accentuating the intricate nature of crystallization phenomena within this system.

### 3.6. Iridescence as a Proof for Grating Assembly in Straight Dendrites vs. Circularly Banded PBA

In addition to the potential interference from top-surface ring bands on thin films, such iridescent properties are also known in some of nature’s mineral crystals, such as moonstone [66], an inorganic compound of sodium/potassium aluminum silicate [(Na/K)AlSi_3_O_8_)], or opals (SiO_2_), which self-assemble and possess an inner microstructure consisting of regular layers of stacked crystalline lamellae. Such alternate-layered and orderly microstructures are seen in nature’s moonstones or opals; analogously, such similar grating assemblies are expected in ring-banded polymer spherulites, as proven in several recent studies [67,68]. Such findings are possible only when the interiors of periodically banded crystals are dissected with the advanced techniques demonstrated in the study to expose the interior architectures, and not by a mere analysis of the superficial top surfaces of periodically banded patterns.

**Figure 13:**
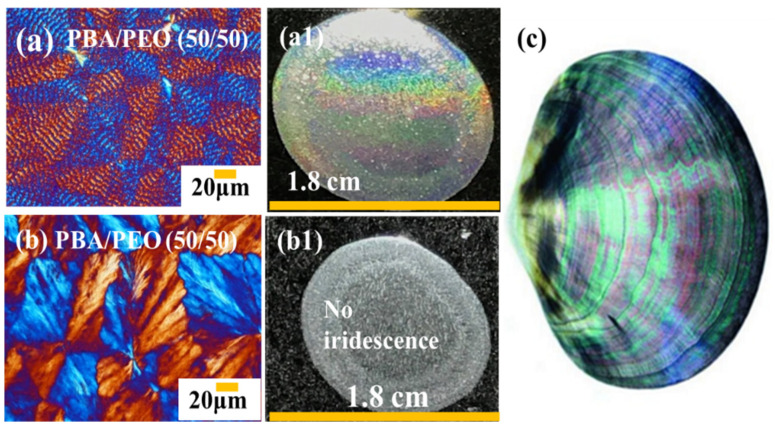
(**a**,**a1**) POM and iridescence from circularly banded PBA; (**b**,**b1**) absence of iridescence in straight dendritic PBA spherulites crystallized from PBA/PEO (50/50) at T_c_ = 30 and 32 °C (reprinted with open access copyrights [63]), respectively; and (**c**) iridescence pattern from the nature’s nacre shell (reprinted/adapted with permission from [69] from © Optical Society of America).

Iridescence tests were performed in line with the similar procedures and experimental setups/apparatus as those used in earlier investigations [62,63]. Figure 13(a,a1) shows striking iridescence from the regularly and circularly banded PBA (T_c_ = 30 °C) spherulites crystallized from a composition of PBA/PEO = 50/50. On the other hand, ringless straight dendritic PBA (at a higher T_c_ = 32 °C), shown in Figure 13(b,b1), crystallized from the same composition [(PBA/PEO) = 50/50] displays no iridescence at all, which is dramatically in contrast with the bright iridescence. This phenomenon is not incidental or sporadic. Conventional circularly-ring-banded spherulites of other polymers are also known to display iridescence features in correlation with the interior orders of lamellae [62,63]. The difference in the banding regularity and, thus, the corresponding iridescence features could be modulated by the mixture composition from which PBA is crystallized. Figure 13c is nature’s iridescence from a nacre shell for side-by-side comparison with that of the PEO-modulated PBA crystals crystallized from PBA/PEO = 50/50 at T_c_ = 30 °C. The iridescence in the circularly banded PBA proves that the lamellae are self-assembled in an orderly, grating-like manner that leads to crystalline and periodically banded aggregates.

## 4. Conclusions

This study provides new insights into the assembly behavior of PBA spherulites, particularly focusing on lamellar assembly transforming from ring-banded to straight dendritic morphologies. The lamellae in either circularly banded or straight dendritic spherulites do not appear to be continuous, nor helix-twisted, like a continuous screw or long DNA molecular helices, all the way from the nucleus center to the periphery. Instead, periodic discontinuities with distinct interfaces are characteristic of the growth cycles that generate optical rings. These growth cycles alternate between perpendicular (tangential) and radial orientations, producing alternating birefringent rings. This behavior has been confirmed through 3D SEM dissection and synchrotron microbeam X-ray analyses.

Synchrotron microbeam X-ray characterization was performed to further analyze the assemblies in the straight dendritic vs. circular-banded zones. For correlation between the top surface and interior structures, the lamellae at the ridge regions are oriented perpendicularly to the substrate. At the same time, they twist by 90° to branch out and form parallel lamellae in the valley regions. This spatial reorganization reflects a dynamic lamellar assembly process not adequately explained by earlier models. Additionally, the irregular twisting and re-branching of lamellae lead to optical extinction bands, whereas the regular arrangement of lamellae results in optical birefringence bands observed in POM. In dendritic spherulites, particularly in the PBA/PEO system with high PEO content and crystallized at elevated temperatures, edge-on lamellae emerge from the nucleus, subsequently bending or twisting to form flat-on lamellae in a cyclic growth pattern. This behavior parallels the ridge–valley growth mechanism observed in ring-banded morphologies, further reinforcing the idea of a shared underlying assembly process.

Importantly, our study shows that lamellar twisting is not a unique feature confined to periodically banded spherulites but can also occur in straight dendritic morphologies under suitable conditions. This broader perspective on lamellar dynamics is a significant departure from conventional interpretations and advances the current understanding of polymer crystallization. Finally, through iridescence imaging, 3D-dissection electron microscopy, and synchrotron microbeam X-ray diffraction, we propose a novel mechanism of lamellar assembly in ring-banded PBA crystal aggregates facilitated by dilution with amorphous constituents. Iridescence tests revealed optical gratings consistent with orderly internal lamellar structures. The presence of iridescence, reminiscent of that observed in nacre or opal, further supports our conclusion that periodicity arises from well-organized internal gratings—not continuous helical twists.

## Data Availability

The original contributions presented in this study are included in the article/supplementary material. Further inquiries can be directed to the corresponding author.

## Supplementary Materials

The following supporting information can be downloaded at https://www.mdpi.com/article/10.3390/polym17152040/s1. Figure S1: Microbeam X-ray 1D-SAXS profiles on 16 spots across alternate RBS and RLS zones. Spots #1–5: ringless, Spots #6–10: ring-banded, Spots #11–16: ringless (#1–16: one complete growth cycle); Figure S2: One-dimensional correlation analysis of SAXS from PBA-RBS at spot #3; lamellae parameters estimated as L_o_ = 10.8 nm, L_c_ = 4.6 nm, L_a_ = 6.2 nm.
