# Supplementary material for: Microbeam X-Ray Investigation of the Structural Transition from Circularly Banded to Ringless Dendritic Assemblies in Poly(Butylene Adipate) Through Dilution with Poly(Ethylene Oxide)
Source: Polymers (Basel). 2025 Jul 26;17(15):2040. doi: 10.3390/polym17152040 (PMC12349629; doi:10.3390/polym17152040)
Supplement: Supplementary file 1 [file polymers-17-02040-s001.zip › polymers-3753472-supplementary.pdf]

Microbeam X-ray Study on Assemblies Transforming from Ring-Banded to Ringless-Dendritic Structures in Poly(butylene adipate) through Dilution with Poly(ethylene oxide)

Selvaraj Nagarajan[1],Chia-I Chang[1], I-Chuan Lin[1], Yu-Syuan Chen[1], Chean-Cheng Su[2], Li-Ting Lee[3,*], and Eamor M. Woo[1,*],

[1] Department of Chemical Engineering, National Cheng Kung University, Tainan, 701-01, Taiwan.
[2] Department of Materials Science and Engineering, Feng Chia University, Taichung, 407-24, Taiwan.
[3] Department of Chemical and Materials Engineering, National University of Kaohsiung, Kaohsiung, 811, Taiwan.

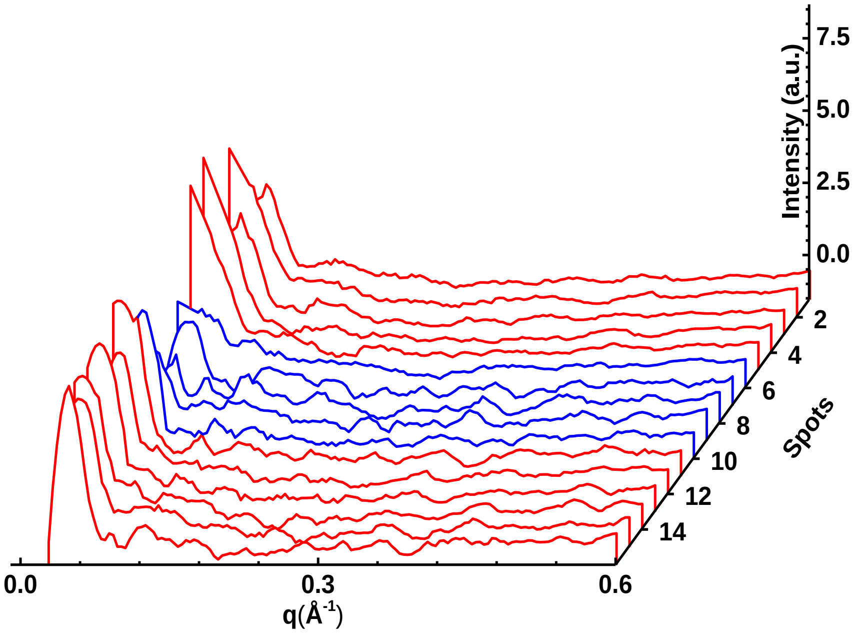

Figure S1: Microbeam X-ray 1D-SAXS profiles on 16 spots across alternate RBS and RLS zones. Spots #1-5: ringless; Spots #6-10: ring-banded; Spots #11-16: ringless (#1-16: one complete growth cycle).

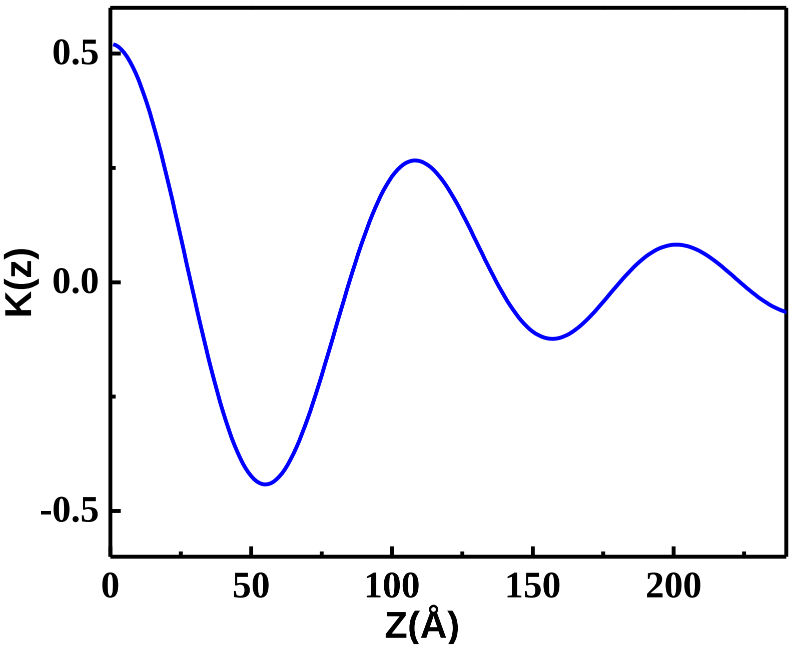

Figure S2:1D-corelation analysis of SAXS from PBA-RBS at spot #3, lamellae parameters estimated as L_o = 10.8 nm, L_c = 4.6 nm, and L_a = 6.2 nm; percentage crystallinity: $L_c / L_o \times 100$.
